# Synergistic Effects of Exercise and Nano-Curcumin Supplementation in Women with Lifestyle-Related Diseases: A Scoping Review

**DOI:** 10.3390/nu17213334

**Published:** 2025-10-23

**Authors:** Nafih Cherappurath, Muhammed Navaf, Halil İbrahim Ceylan, Masilamani Elayaraja, Kappat Valiyapeediyekkal Sunooj, Saranya T. Satheesan, Muhammed Ali Thoompenthodi, Shamshadali Perumbalath, Serdar Bayrakdaroğlu, Raul Ioan Muntean, Nikolaos Mavritsakis, Dilshith A. Kabeer

**Affiliations:** 1 Department of Physical Education, Amal College of Advanced Studies (Autonomous), Nilambur 679329, India; nafihch@amalcollege.ac.in; 2 Department of Food Science and Technology, Pondicherry University, Puducherry 605014, India; navafmak@gmail.com (M.N.); sunooj4u@gmail.com (K.V.S.); 3 Faculty of Sports Sciences, Atatürk University, Erzurum 25100, Türkiye; 4 Department of Physical Education and Sports, Pondicherry University, Puducherry 605014, India; elaya.cricket@gmail.com (M.E.); dilshid7@gmail.com (D.A.K.); 5 Amity Institute of Behavioural Health and Allied Sciences, Amity University, Bangalore 562110, India; saranya.t.sathish@gmail.com; 6 Department of Physical Education, Malabar College of Advanced Studies, Vengara 676519, India; alimohammedkpz@gmail.com; 7 Department of Banking Technology, Pondicherry University, Puducherry 605014, India; shamshadali.nbr@pondiuni.ac.in; 8 Faculty of Sports Sciences, Gümüşhane University, Gumushane 29000, Türkiye; bayrakdaroglu85@gmail.com; 9 Faculty of Law and Social Sciences, University “1 Decembrie 1918” of Alba Iulia, 510009 Alba Iulia, Romania

**Keywords:** nano-curcumin, phytochemicals, exercise, women’s health, lifestyle-related diseases, metabolic disorders, antioxidants, inflammation

## Abstract

**Background/Objectives:** Lifestyle-related diseases such as obesity, diabetes, hypertension, metabolic syndrome, non-alcoholic fatty liver disease (NAFLD), and osteoarthritis disproportionately affect women due to hormonal, metabolic, and socio-cultural factors. Emerging evidence suggests that combining structured exercise with nano-curcumin, a bioavailable phytochemical formulation with potent antioxidant and anti-inflammatory properties, may provide synergistic benefits. This scoping review systematically synthesizes available evidence on the combined effects of nano-curcumin supplementation and exercise interventions on health outcomes in women with lifestyle-related diseases. **Methods:** Following the Joanna Briggs Institute methodology and the PRISMA-ScR framework, a comprehensive database search was conducted in March 2025 and updated in June 2025. Records were retrieved from Scopus (n = 30), Web of Science (n = 22), PubMed (n = 18), and other sources (n = 71), yielding a total of 141 studies. After screening and deduplication, eight studies met the inclusion criteria and were included in this review. All the studies were conducted in Iran with small sample sizes (12–53 participants) and short intervention durations (6–16 weeks). Therefore, the current evidence is geographically and demographically limited. **Results:** Across the included trials, the combined interventions produced additive or synergistic improvements in oxidative stress markers, inflammatory cytokines, lipid and glucose metabolism, cardiovascular function, pulmonary capacity, muscle fitness, and psychological outcomes (e.g., depression). When paired with nano-curcumin supplementation at different concentrations, high-intensity interval training, aerobic exercise, Pilates, and resistance training consistently outperformed exercise or supplementation alone in modulating antioxidant defenses, reducing systemic inflammation, and improving metabolic risk factors. **Conclusions:** The integration of exercise and nano-curcumin supplementation appears to confer superior benefits for women with lifestyle-related diseases compared with either approach alone. These findings highlight the potential of combining phytochemicals with lifestyle interventions to optimize women’s health outcomes. However, most available evidence originates from small, short-term studies in single geographic regions. Large-scale, multicenter, randomized controlled trials with diverse populations are warranted to establish standardized protocols and optimal dosing strategies, and to assess long-term safety and efficacy.

## 1. Introduction

Lifestyle-related diseases such as obesity, cardiovascular disorders, metabolic syndrome, and type 2 diabetes are increasing worldwide, with women disproportionately affected due to hormonal, metabolic, and socio-cultural factors [1]. These non-communicable diseases are strongly associated with sedentary behaviours, poor dietary habits, hormonal imbalance, and chronic low-grade inflammation [2]. The World Health Organization (WHO) estimates that non-communicable diseases, like lifestyle diseases, account for 70% of all deaths worldwide, with women being disproportionately affected by these conditions due to gender-specific risk factors and metabolic variations [3]. The increase in disease burden and reduced quality of life among individuals with lifestyle diseases, particularly women, requires integrative therapeutic strategies that address both the physiological and behavioral dimensions of these conditions.

Regular exercise is a cornerstone in preventing and managing lifestyle-related diseases, as it enhances cardiovascular function, regulates lipid metabolism, and improves hormonal balance while also reducing oxidative stress and inflammation. Regular physical activity helps improve cardiovascular health, lipid profile, and hormonal regulation while reducing inflammation and oxidative stress [4,5]. Although it has numerous benefits, adherence to exercise regimes remains low among women due to socio-cultural, psychological, and logistical barriers [6]. This suggests the potential benefit of adjunct therapies, such as dietary supplementation, to enhance the effects of exercise and provide additional motivation and health benefits.

Curcumin, a polyphenolic compound derived from *Curcuma longa*, demonstrates potent antioxidant, anti-inflammatory, and metabolic regulatory properties. However, due to its low bioavailability, its clinical application has been limited. Its low bioavailability restricts its extensive use in therapeutic applications. Recent advances in nanotechnology have enabled the synthesis of nano-curcumin formulations. Nano-curcumin can be developed using diverse techniques, such as nanoprecipitation, solvent evaporation, single emulsion, spray drying, microemulsion, and emulsion polymerization. Nano-formulations of curcumin have significant advantages; they exhibit enhanced curcumin solubility, stability, and absorption [7]. This facilitates augmented cellular uptake and biological response, thereby amplifying the therapeutic potential of curcumin, as reported by Hassanizadeh et al. [8] and Yallapu et al. [9].

A promising approach to achieving synergistic health benefits in women with lifestyle diseases, metabolic disorders, and inflammatory disorders is the incorporation of nano-curcumin supplementation with exercise. It is hypothesized that the synergistic effect of exercise and nano-curcumin overlaps several metabolic pathways that regulate oxidative stress and inflammation, etc. Exercise may induce mild oxidative stress, while nano-curcumin further enhances these mechanisms due to its appreciable bioactivity. Hence, the synergistic application of these exercises and nano-curcumin may enhance cellular mechanisms to a greater extent than alone. This scoping review aims to compile and structure existing scientific data about the synergistic benefits of exercise and nano-curcumin supplementation on the health outcomes of women with lifestyle-related diseases.

## 2. Methods

This scoping review aimed to synthesize empirical evidence on the combined effects of exercise and nano-curcumin supplementation on women’s health with lifestyle-related diseases. This review followed the method laid out by the Joanna Briggs Institute (JBI) [10] and the structure recommended by Arksey and O’Malley [11], which includes the following stages: (i) formulation of the research question, (ii) identification of relevant studies, (iii) study selection, (iv) data extraction, and (v) collation, summarization, and reporting of results. This review also followed the Preferred Reporting Items for Systematic Reviews and Meta-Analyses extension for Scoping Reviews (PRISMA-ScR) checklist (Supplementary File S1) to ensure that the methods were clear and that all the information was included [12]. The review protocol was developed and registered on the Open Science Framework (OSF) to ensure transparency and methodological rigor (https://doi.org/10.17605/OSF.IO/3G5TK (accessed on 16 October 2025)).

### 2.1. Information Sources and Search Strategy

**Figure 1:**
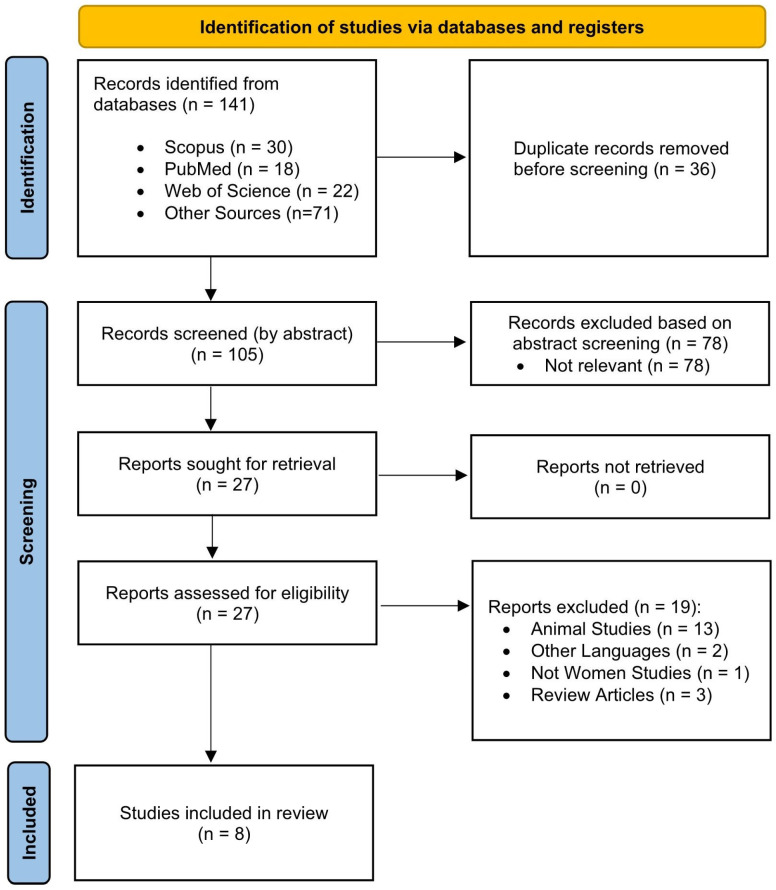
PRISMA flow diagram illustrating the study selection process.

The initial data search was conducted in March 2025; an updated search was performed in June 2025. This process employed four electronic databases that were designed explicitly for the study of sports science and medicine: (i) Scopus, (ii) Web of Science, (iii) PubMed, and (iv) Google Scholar. The results of the literature search were manually reviewed to identify duplicate publications. The search strategy combined “exercise” and “nano-curcumin” to target women with lifestyle diseases. The search was conducted using a Boolean technique with the OR operator to combine the following keywords: (“physical activity” OR “exercise” OR “training”) AND (“nano-curcumin” OR “nano curcumin” OR “nanocurcumin”) AND (“women” OR “female” OR “girl”). Two independent reviewers (NC and MN) screened the selected articles using the inclusion criteria. The screening procedure had two phases: (1) the screening of the title and abstract, and (2) reviewing the complete paper. Duplicates were eliminated. Discussions regarding any disagreements took place following each stage. A review of the reference lists from the chosen research was also conducted to identify other relevant papers. At each step of the selection process, a PRISMA flow diagram (Figure 1) shows which studies were considered, rejected, and ultimately included.

### 2.2. Eligibility Criteria

This study’s inclusion and exclusion criteria aligned with the PICOS framework (Intervention, Comparison, Outcome, and Study design) as provided in Table 1 [13].

### 2.3. Critical Appraisal of the Articles

The risk of bias in randomized controlled trials was assessed using the JBI Critical Appraisal Tool for Randomized Controlled Trials. For non-randomized controlled trials, the JBI checklist for quasi-experimental designs was applied. The proportion of affirmative (“yes”) responses was used to determine the overall risk of bias. Studies achieving a score of ≥70% were classified as having a low risk of bias, those scoring between 50% and 69% as having a moderate risk of bias, and those scoring < 50% as having a high risk of bias [14,15].

## 3. Results

### 3.1. Search Results

Initially, 141 manuscripts were identified through database searches (Scopus, 30; Web of Science, 22; PubMed, 18) and other sources (n = 71). After excluding 36 duplicate records, 105 were screened at the title and abstract level, and 78 studies were excluded. Of the 27 reports requested for full-text retrieval, one was unavailable, leaving 26 to be evaluated for eligibility. Nineteen papers were rejected following a comprehensive examination for the following reasons: animal studies (n = 13), non-English language (n = 2), not specific to women (n = 1), and review articles (n = 2). Eight studies met all inclusion criteria and were included in the final review (Figure 1).

### 3.2. Characteristics of the Study and Participants

Table 2 provides a detailed summary of the studies included and the characteristics of the participants. Data extraction from each study was organized according to the PICOS framework. The studies were conducted in Iran from 2019 to 2024, employing various experimental designs, including randomized controlled trials, non-randomized controlled trials, placebo-controlled trials, pre-post intervention studies, and open-label parallel designs. The sample sizes ranged from 12 to 53 participants. This study only looked at women who had conditions related to their lifestyle, such as overweight teenage girls, women with metabolic syndrome, women over 25 with non-alcoholic fatty liver disease (NAFLD), women with knee osteoarthritis, and overweight older women who did not have any other health problems. Nano-curcumin supplementation and structured exercise interventions, including high-intensity interval training, aerobic exercise, Pilates, resistance training, and Tabata training, were evaluated in relation to lifestyle disease outcomes in all studies.

### 3.3. Interventions

All included studies focused on the synergistic effects of nano-curcumin supplementation and exercise interventions on lifestyle diseases. The nano-curcumin dosage was 80 mg/day in six studies [16,17,18,19,22,23], while two studies reported a higher dose of 1000 mg/day [20,21]. Considerable variation was observed across studies regarding exercise modality, intensity, duration, and delivery. Fakhri et al. studied antioxidant capacity and lipid metabolism in overweight teenage girls [16] after they completed a 6-week high-intensity interval training program combined with nano-curcumin supplementation. The effects of six weeks of moderate-intensity aerobic exercise supplemented with nano-curcumin on BDNF and inflammatory markers (IL-6 and IL-10) in patients with metabolic syndrome aged 60 to 65 years were also studied by Osali [17]. Osali and Rostami [18] further examined the impact of a 6-week moderate aerobic training program with nano-curcumin on pro-inflammatory cytokines (IL-1β, NO) and depressive symptoms in women with metabolic syndrome in the same age group.

Rezaei et al. [19] conducted an additional investigation to evaluate the combined effects of nano-curcumin supplementation and Pilates training on overweight and obese women diagnosed with NAFLD. Cheragh-Birjandi et al. [21] investigated the impact of nano-curcumin resistance training on synovial collagenase-2 and nitric oxide (NO) levels in women with knee osteoarthritis. In 2023, their subsequent study expanded upon this work by analyzing the impacts of the same intervention conditions on cartilage biomarkers, specifically matrix metalloproteinase-13 (MMP-13) and COMP. In obese menopausal women aged 40–60, Dabidi Roshan et al. [22] conducted an eight-week HIIT program with low or moderate training volumes and nano-curcumin supplementation. They assessed circulatory hemodynamics, pulmonary function, muscle fitness, and physical performance. Finally, Noorbakhsh and Roshan [23] examined the individual and synergistic effects of eight weeks of Tabata-style HIIT and nano-curcumin supplementation on NLRP3 inflammasome activity, MIAT expression, body composition, and cardiorespiratory fitness in overweight older women.

### 3.4. Study Outcomes

The eight included studies employed diverse outcome measures, reflecting variations in the specific lifestyle diseases and training protocols investigated. In an inquiry of overweight and obese women aged 25 years and older who were diagnosed with NAFLD, Rezaei et al. [19] studied the impact of eight weeks of Pilates training in conjunction with nano-curcumin supplementation on anthropometric parameters, including height, weight, body mass index (BMI), waist-to-hip ratio (WHR), and thigh circumference, as well as hepatic steatosis—a similar study by Fakhri et al. [16] examined the effects of six weeks of high-intensity interval training combined with nano-curcumin on oxidative stress biomarkers in overweight schoolgirls. The biomarkers included malondialdehyde (MDA), glutathione peroxidase (GPX), glutathione (GSH), catalase (CAT), and superoxide dismutase (SOD).

The combined benefits of six weeks of aerobic exercise and nano-curcumin supplementation in women aged 60–65 with metabolic syndrome were examined by Osali [17] and Osali & Rostami [18]. The outcomes studied included plasma concentrations of NO, depression ratings, brain-derived neurotrophic factor (BDNF), interleukin-1β, interleukin-6, TAC, and MDA. Cheragh-Birjandi et al. [20,21] evaluated the synergistic effects of 16 weeks of resistance exercise and nano-curcumin supplementation on biochemical markers of knee osteoarthritis. Specifically, they examined collagenase-II and NO levels in synovial tissue, matrix metalloproteinase-13 (MMP-13) concentrations in synovial fluid, and COMP concentrations in synovial fluid.

Dabidi Roshan et al. [22] assessed the effects of eight weeks of low- versus moderate-volume HIIT, with or without nano-curcumin supplementation, in obese menopausal women aged 45–60 years. Their findings indicated notable enhancements in cardiovascular hemodynamics, pulmonary function, and muscular fitness, as well as improvements in physical performance metrics including blood oxygen saturation, heart rate (HR), systolic blood pressure (SBP), diastolic blood pressure (DBP), maximal oxygen uptake (VO_2_max), forced vital capacity (FVC), forced expiratory volume in one second (FEV_1_), forced expiratory flow (FEF), and peak expiratory flow (PEF), along with observed changes in muscle strength. Noorbakhsh and Roshan [23] examined the impact of eight weeks of Tabata-style high-intensity interval training alongside nano-curcumin supplementation on body composition, cardiorespiratory fitness, and molecular markers, specifically the expression of long non-coding RNA myocardial infarction-associated transcript (lncRNA MIAT) and NOD-like receptor family pyrin domain-containing 3 (NLRP3) inflammasome activity, in overweight older women.

### 3.5. Key Findings

Across the reviewed studies, the results consistently indicated that the synergistic combination of nano-curcumin supplementation and physical exercise produced favorable outcomes in patients with various lifestyle-related diseases (Table 3). Fakhri et al. [16] reported that in overweight adolescent girls, the combined intervention of HIIT and nano-curcumin supplementation significantly increased CAT (*p* = 0.001), GSH (*p* = 0.006), SOD (*p* = 0.015), and GPX (*p* = 0.05) levels while markedly reducing MDA concentrations (*p* = 0.009). In contrast, HIIT alone was associated with a significant rise in serum MDA (*p* = 0.004) but did not significantly change SOD, GPX, CAT, or GSH. At the same time, nano-curcumin supplementation alone increased GSH (*p* = 0.001), SOD (*p* = 0.006), and CAT (*p* = 0.01), while GPX and MDA remained unchanged. Simultaneous use of nano-curcumin and aerobic exercise decreased serum MDA and hs-CRP levels while increasing BDNF, interleukin-10 (IL-10), and total antioxidant capacity in women with metabolic syndrome [17]. In a similar vein, Osali and Rostami [18] observed that combining aerobic exercise with nano-curcumin supplementation resulted in a significant increase in interleukin-1β (IL-1β) levels (4.99) compared with the control group (4.87). In contrast, either exercise or supplementation alone produced values of 4.80 and 4.77, respectively, lower than the control group’s IL-1β value of 4.87. NO concentrations also increased across all intervention groups relative to the control group, with the combined training and supplementation group showing the highest values. This combined intervention was associated with reductions in depressive symptoms, a trend also observed in the exercise-only and supplementation-only groups, with a greater impact in the combined group.

Rezaei et al. [19] found that in obese women, Pilates training combined with nano-curcumin supplementation significantly enhanced biological markers, including alanine transaminase, Aspartate transaminase, and alkaline phosphatase, compared with the training group. Interestingly, Alanine transaminase was decreased when training alone was performed.

Cheragh-Birjandi et al. [21] reported that, in women with knee osteoarthritis, both resistance training alone and in combination with nano-curcumin (1000 mg/day) supplementation increased collagenase II activity compared with the control group, with the highest activity observed in the training-only group. In contrast, collagenase II activity decreased in the group receiving nano-curcumin supplementation alone. Synovial NO levels were reduced across all study groups compared with the control.

Nonetheless, a later investigation by the same group [20] revealed that exercise combined with supplementation had minimal effects on MMP13 after a 16-week schedule, whereas MMP13 was decreased in the control and separate training and supplementation groups compared with pre-test values. Likewise, compared with pre-test values, COMP values also decreased after 16 weeks of training across all groups, with the smallest reduction observed in the supplementation group, followed by the combined training and supplementation group, and the most significant decrease in the control group.

Ultimately, Dabidi Roshan et al. [22] indicated that moderate-volume HIIT yielded mixed outcomes in obese menopausal women. Pulmonary function metrics, such as forced vital capacity (FVC), forced expiratory volume in 1 s (FEV1), forced expiratory flow (FEF), and peak expiratory flow (PEF), exhibited a decline. At the same time, cardiovascular parameters showed enhancement, characterized by elevations in heart rate and maximal oxygen uptake (VO_2_max) and decreases in both systolic and diastolic blood pressure.

### 3.6. Critical Appraisal of the Studies

Among the six randomized controlled trials assessed, three —Rezaei et al. [19], Dabidi Roshan et al. [22], and Noorbakhsh & Roshan [23]—achieved scores of 77%, indicating a low risk of bias. The remaining three studies, like those of Osali [17], Cheragh-Birjandi et al. [21], and Cheragh-Birjandi et al. [20], scored between 62% and 69%, reflecting a moderate risk of bias. Two quasi-experimental studies were evaluated: Osali & Rostami [18] scored 78%, indicating a low risk of bias, whereas Fakhri et al. [16] scored 67%, indicating a moderate risk of bias. Across all studies, the majority (four out of eight) demonstrated a low risk of bias, while the remaining four showed a moderate risk. No study was classified as having a high risk of bias (Table 4).

## 4. Discussion

This scoping review synthesized available evidence on the combined effects of exercise and nano-curcumin supplementation on health outcomes in women with lifestyle-related diseases, including overweight/obesity, NAFLD, metabolic syndrome, and knee osteoarthritis. Regarding the included studies, combining structured exercise with nano-curcumin supplementation consistently yielded additive or synergistic benefits, especially in lowering systemic inflammation, boosting antioxidant defenses, improving cardiometabolic health, and easing musculoskeletal symptoms in women with lifestyle-related disorders. All evidence came from studies in a single geographic area, focusing exclusively on women with one or more of five lifestyle-related conditions.

The analyzed research indicated that, regardless of physical activity, nano-curcumin supplementation significantly impacted numerous health outcomes. These effects are primarily attributed to its bioactive properties, particularly its antioxidant capacity. Curcumin, a polyphenol derived from plants, exhibits significant antioxidant properties by mitigating oxidative stress and preventing the generation of free radicals, with its effectiveness claimed to surpass that of vitamin E [24]. Curcumin enhances endogenous antioxidant defenses by upregulating GSH, CAT, and SOD, thereby neutralizing reactive oxygen species (ROS) [25]. It also inhibits the activity of ROS-generating enzymes, such as lipoxygenase, cyclooxygenase, and xanthine oxidase/hydrogenase [26]. Nano-curcumin supplementation, with and without HIIT, for instance, dramatically raised GPX, GSH, CAT, and SOD levels in overweight female students while lowering MDA concentrations [16].

The evaluated studies also addressed metabolic syndrome, a collection of interrelated metabolic abnormalities that elevate the risk of cardiovascular disease (CVD) and type 2 diabetes mellitus (T2DM). The evidence suggests that the synergistic effects of aerobic exercise and nano-curcumin supplementation may ameliorate depressive symptoms in women aged 60–65 years who have metabolic syndrome. This effect is thought to occur through a combination of higher HDL cholesterol levels and decreases in IL-1β, blood pressure, triglycerides, and waist circumference [18]. Lowering IL-1β levels may enhance serotonin modulation, thereby reducing depressive symptoms [27]. Curcumin can also reduce systemic inflammation by decreasing CRP and IL-6 levels [28]. These findings highlight the anti-inflammatory properties of nano-curcumin when combined with exercise therapy.

Nano-curcumin reduces inflammation through various mechanisms, including regulating inflammatory processes in adipose tissue. Shehzad et al. [29] found that it inhibits NF-κB, a transcription factor that regulates cytokine gene expression. It also inhibits pro-inflammatory intracellular signaling kinases and decreases macrophage infiltration into adipose tissue, lowering pro-inflammatory cytokine production and alleviating obesity-related inflammation and metabolic dysfunction [30].

This review also examined osteoarthritis, a degenerative joint disease characterized by the progressive deterioration of cartilage, which is associated with pain, stiffness, and reduced mobility. The pathophysiology of cartilage degradation is partially influenced by elevated levels of collagenase-2, NO, and matrix metalloproteinase-13 (MMP-13) in the synovium and synovial fluid [31,32]. According to clinical studies, resistance training combined with nano-curcumin supplementation decreased collagenase-2 and NO levels in women with knee osteoarthritis [21]. In contrast, Cheragh-Birjandi et al. [20] found no statistically significant changes in COMP or MMP-13 concentrations when treated with supplements. There were some improvements in pain and stiffness, but these were not the main goals of the therapies.

Other evidence suggests that the cardiometabolic benefits of combining nano-curcumin with exercise are notable. Noorbakhsh and Roshan [23] demonstrated that supplementation with nano-curcumin alongside Tabata-style HIIT improved body composition, cardiorespiratory fitness, and cardiovascular function in overweight older women. While there were no discernible impacts on lncRNA-MIAT expression, notable results included enhanced oxygen consumption, elevated VO_2_max, decreased resting heart rate, systolic and diastolic blood pressure, and NLRP3 inflammasome activity.

This scoping review highlights that integrating nano-curcumin supplementation with structured exercise interventions confers positive health effects in women with lifestyle-related conditions, acting through antioxidant, anti-inflammatory, and metabolic pathways. The comprehensive search across four databases without temporal restrictions ensured broad coverage of the available literature. To our knowledge, this is the first scoping review to systematically investigate the synergistic impact of nano-curcumin supplementation and exercise in this population.

Preliminary evidence suggests that combined exercise and nano-curcumin interventions may affect specific health outcomes in women with lifestyle-related conditions. However, current data are insufficient to support clinical recommendations. Future research should focus on well-designed, adequately powered randomized controlled trials that include diverse populations and report detailed nano-curcumin characterization for meaningful comparison and synthesis.

### 4.1. Study Limitations

This study has several limitations. First, all eight studies were conducted in Iran, involving relatively small sample sizes and brief intervention periods. As a result, the findings cannot be generalized to women with lifestyle-related diseases in other regions or populations. These limitations underscore the urgent need for larger, multicenter randomized controlled trials to assess the combined effects of exercise and nano-curcumin supplementation across diverse settings and populations. The included studies show significant variation in intervention type, exercise intensity, and nano-curcumin formulations, such as dosage. While some studies reported improvements in markers like oxidative stress, inflammatory biomarkers, cardiometabolic parameters, and musculoskeletal symptoms, these outcomes were inconsistent across studies. Additionally, the results varied depending on the exercise protocol and study population. Second, the research was limited to articles retrieved from electronic databases, and only full-text papers were included, excluding abstracts and conference proceedings. Third, the search was confined to English-language publications, potentially omitting relevant studies in other languages. Considerable variability in exercise protocols and nano-curcumin formulations further limits the standardization and comparability of these studies. The study populations lacked diversity, with limited representation across ethnic groups and age ranges. Although information on exercise modalities and outcome measures was available, most included studies lacked detailed reporting of nano-curcumin particle size and bioavailability, limiting the depth of analysis in this review.

### 4.2. Future Directions

Future research should conduct large-scale, multi-center, global randomized controlled trials to further validate the combined benefits of organized exercise and nano-curcumin supplementation across populations with diverse genetic backgrounds, cultural contexts, and geographic locations. These studies need to address key questions, such as the duration of effects, optimal dosage, and timing relative to physical activity. Thorough safety monitoring, including assessment of potential drug-nutrient interactions and long-term follow-up, is essential to determine whether effects persist beyond 12 weeks. Future research should also explore the molecular mechanisms underlying the observed synergy to inform targeted therapies better. Additionally, comprehensive assessments of quality-of-life outcomes, cost-effectiveness analyses, and implementation research within various healthcare systems are warranted. These efforts will strengthen the evidence base, improve clinical relevance, and broaden the applicability of recommendations for women with lifestyle-related disorders.

None of the included studies provided detailed data on adverse events, biochemical abnormalities, or participant discontinuations related to nano-curcumin supplementation or its combination with exercise. This lack of safety reporting highlights a significant gap in the current literature. Although curcumin is generally considered safe at moderate doses, there are known risks of hepatotoxicity, gastrointestinal discomfort, and drug–nutrient interactions, especially with anticoagulants and chemotherapeutic agents. Therefore, the safety profile of nano-curcumin formulations—due to their increased bioavailability—should be thoroughly evaluated in future studies.

## 5. Conclusions

This scoping review shows that combining nano-curcumin supplementation with structured exercise programs provides greater benefits for women with lifestyle-related disorders than using either strategy alone. The evidence from the included studies highlights improvements in oxidative stress, systemic inflammation, cardiometabolic risk factors, and physical performance. While these results are encouraging, the current evidence is limited by small sample sizes, short intervention periods, and limited geographic diversity. To advance this field, future large-scale, multicenter randomized controlled trials should develop standardized protocols to determine the optimal dosing, duration, and timing of nano-curcumin in combination with exercise. This research is crucial to confirm long-term safety and effectiveness, improve generalizability, and turn this combined approach into practical strategies for enhancing women’s health outcomes.

## Figures and Tables

**Table 1:** Eligibility of the studies based on the PICOS framework.

Category	Inclusion Criteria	Exclusion Criteria
Population	Women (any age group) diagnosed with one or more lifestyle-related diseases.	Studies on men only, or mixed-gender studies without sex-specific data for women Studies on animals or in vitro (cell-based) research
Intervention	Studies that involve exercise (any type: aerobic, resistance, Pilates, etc.) and nano-curcumin supplementation Interventions may be combined or administered separately, as long as both are part of the study design.	Studies involving only exercise or nano-curcumin, with no combination of both. Studies that use other curcumin formulations (e.g., turmeric extract, curcuma longa powder) without specifying “nano-curcumin”.
Comparison	Studies that provided pre-to-post only (no control) comparisons or included a control condition	Studies lacking any comparator or baseline-to-post measurements (i.e., descriptive or observational without intervention effect measurement) Studies comparing unrelated interventions not involving exercise or nano-curcumin
Outcomes	Studies reporting at least one relevant health-related outcome (e.g., metabolic biomarkers, inflammatory markers, hormonal profiles, body composition or weight-related outcomes, quality of life, mental health, or physical performance measures).	Studies that do not measure or report human health outcomes (e.g., formulation studies, pharmacokinetics only) Articles focusing solely on mechanisms or molecular pathways without clinical or human health outcomes.
Study design	Peer-reviewed original research articles, including: Quasi-experimental studies Randomized controlled trials Cohort or longitudinal studies Studies published in English	Reviews, meta-analyses, editorials, commentaries, protocols, or conference abstracts Non-English publications Studies not available in full text

**Table 2:** Study characteristics.

Sl. No.	Author Name and Year	Country	Study Design	Objective	Sample Size and Population
1	Fakhri et al. (2020) [16]	Iran	Pre and post design	To examine the impact of six weeks of high-intensity interval training, supplemented with nano-curcumin, on overweight females’ antioxidant defense and lipid breakdown.	48 overweight female students
2	Osali (2020) [17]	Iran	Double-blind, placebo-controlled, and semi-experimental design	To examine the effect of a 6-week moderate-intensity aerobic exercise program and nano-curcumin supplementation on IL-6, IL-10, and BDNF levels in females aged 60 to 65 with metabolic syndrome.	44 women aged 60–65 with metabolic syndrome
3	Osali and Rostami (2023) [18]	Iran	Randomized controlled trial	To examine the impact of six weeks of moderate-intensity aerobic exercise and nano-curcumin consumption on IL-1β, nitric oxide (NO), and depression in women aged 60 to 65 with metabolic syndrome.	44 women aged 60–65 with metabolic syndrome
4	Rezaei et al. (2023) [19]	Iran	Randomized, single-blinded, placebo-controlled trial	To examine the effects of Pilates training and nano-curcumin on overweight and obese women with NAFLD.	12 females with NAFLD over 25 years of age
5	Cheragh-Birjandi et al. (2022) [20]	Iran	Randomized trial study	To examine the impact of resistance training and nano-curcumin supplementation on matrix metallopeptidase 13 (MMP13) and cartilage oligomeric matrix protein (COMP) in people with knee osteoarthritis.	40 women aged 45–60 years with knee osteoarthritis.
6	Cheragh-Birjandi et al. (2020) [21]	Iran	Open-label, parallel randomized trial	To determine the impact of resistance training and Nano-curcumin supplementation on synovial collagenase-2 and nitric oxide levels in people with knee osteoarthritis.	40 women aged 50–65 years with primary knee osteoarthritis.
7	Dabidi Roshan et al. (2024) [22]	Iran	Double-blind, randomized, and placebo-controlled design	To determine which nonpharmacological approach (8-week low-volume vs. moderate-volume-HIIT with or without NaC supplementation) affects the cardiovascular hemodynamic, pulmonary function, muscular fitness, and body performance in obese menopausal women aged 45–60 years.	53 women aged 45–60 years with obese menopause, with an average.
8	Noorbakhsh and Roshan (2023) [23]	Iran	Double-blind, randomized, placebo-controlled trial	To examine the effects of 8 weeks of Tabata-HIIT and NaC supplementation (individual and combined) on the NOD-like receptor family pyrin domain-containing 3 (NLRP3) inflammasome, lncRNA MIAT expression, body composition, and cardiorespiratory health in elderly overweight women.	48 healthy overweight elderly women

**Table 3:** Effect of nano-curcumin supplementation on the health outcome of women.

Sl. No.	Authors and Year	Study Type	Type of Physical Activity/Exercise	Duration	Dosage of Nano-Curcumin (mg)	Outcome Measures	Key Findings
1	Fakhri et al. (2020) [16]	Semi-experimental Study	High-intensity interval training	6 weeks	80	measure the MDA, GPX, glutathione, CAT, and SOD	A significant increase in serum MDA levels (*p* = 0.004) was observed in the training group after six weeks. In the supplement group, serum levels of GSH (*p* = 0.001), SOD (*p* = 0.006), and CAT (*p* = 0.01) increased significantly. Furthermore, in the combined training and supplementation group, significant increases were observed in CAT (*p* = 0.001), GSH (*p* = 0.006), SOD (*p* = 0.015), and GPX (*p* = 0.05) levels, along with a significant decrease in MDA (*p* = 0.009).
2	Osali (2020) [17]	Double-blind, placebo-controlled, and semi-experimental design	Aerobic exercise training with a treadmill, with 5 min rest periods between the sets	6 weeks	80	IL-6, IL-10 and BDNF	Concentrations of IL-10 and BDNF significantly increased following the 6-week intervention (*p* ≤ 0.05), while serum IL-6 levels significantly decreased (*p* ≤ 0.05). Furthermore, the findings indicate that nano-curcumin supplementation significantly reduced serum concentrations of MDA and high-sensitivity C-reactive protein (hs-CRP) in individuals with metabolic syndrome.
3	Rezaei et al. (2023) [19]	semi-experimental, placebo-based study	Pilates training	8 weeks	80	anthropometric measurements included height, weight, BMI, WHR, and thigh circumference, hepatic steatosis	The WHR (*p* = 0.002) and LDL/HDL ratio (*p* = 0.010) significantly improved in participants who received nano-curcumin supplementation alongside the Pilates protocol. However, Pilates training alone, independent of supplementation, also resulted in significant improvements, including reduced hepatic steatosis, decreased GGT levels, and increased HDL concentrations (*p* < 0.05).
4	Osali and Rostami (2023) [18]	Randomized controlled trial	Aerobic exercise	6 weeks	80	antioxidant indicators and lipid degradation measurement, levels of anxiety	Significant changes in IL-1β, nitric oxide, and depression scores were observed before and after exercise across all three experimental groups (*p* < 0.05). Additionally, significant differences in NO levels and depression were observed among the experimental groups, with the most significant reductions occurring in the training and training plus supplementation groups (*p* < 0.05).
5	Cheragh-Birjandi et al. (2022) [20]	Randomized trial study	Resistance training	16 weeks	1000	The change in the synovial fluid levels of MMP-13 and COMP	Resistance training, supplement intake, and combined intervention did not significantly change synovial COMP or MMP-13 levels. However, compared to the control group, WOMAC scores were significantly higher in the intervention groups (*p* = 0.038).
6	Cheragh-Birjandi et al. (2020) [21]	Open-label, parallel randomized trial	Resistance Exercise	16 weeks	1000	synovial level of collagenase-II and NO	No significant correlations were found between resistance exercise, nano-curcumin supplementation, and synovial collagenase-2 or nitric oxide levels in women with knee osteoarthritis. However, both markers showed reduced levels following supplementation, suggesting a potential modulatory effect of nano-curcumin.
7	Dabidi Roshan et al. (2024) [22]	Double blind, randomized, placebo-controlled study	Low-volume-HIIT and moderate-volume-HIIT	8 weeks	80	Cardiovascular dynamics, respiratory function, and physical performance	After 8 weeks of LV-HIIT and MV-HIIT interventions, maximal exercise testing revealed significant increases in VO2max and oxygen pulse. Furthermore, MV-HIIT, both with and without nano-curcumin supplementation, led to notable improvements in muscular fitness, physical performance, quadriceps strength, sit-to-stand performance, and running distance compared to baseline.
8	Noorbakhsh and Roshan (2023) [23]	Double-blind, randomized, placebo-controlled trial	Tabata High-Intensity Interval Training	8 weeks	80	NOD-like receptor family pyrin domain-containing 3 (NLRP3) inflammasome, long non-coding RNA myocardial infarction-associated transcript (lncRNA MIAT) expression, body composition, and cardiorespiratory health.	Significant improvements in body composition and cardiorespiratory hemodynamics were observed in the Tabata-HIIT groups compared with the nano-curcumin-only and placebo groups (*p* < 0.05). However, Tabata training, with or without nano-curcumin supplementation, did not significantly affect resting lncRNA-MIAT expression levels (*p* > 0.05). Notably, nano-curcumin supplementation combined with Tabata training significantly reduced NLRP3 inflammasome levels, suggesting a potential synergistic anti-inflammatory effect.

**Table 4:** JBI Critical Appraisal for risk of bias assessment.

Sl. No.	Author; Year	Total Responses of ‘Yes’	Percentage	Risk of Bias
Randomized Controlled Trials (No. of Questions = 13)				
1	Osali (2020) [17]	8	62%	Moderate
2	Rezaei et al., 2023 [19]	10	77%	Low
3	Cheragh-Birjandi et al., 2022 [20]	9	69%	Moderate
4	Cheragh-Birjandi et al., 2020 [21]	8	62%	Moderate
5	Dabidi Roshan et al., 2024 [22]	10	77%	Low
6	Noorbakhsh & Roshan (2023) [23]	10	77%	Low
Non-Randomized Controlled Trials (No. of Questions = 9)				
1	Osali & Rostami (2023) [18]	7	78%	Low
2	Fakhri et al. (2020) [16]	6	67%	Moderate

## Data Availability

Not applicable.

## Supplementary Materials

The following supporting information can be downloaded at: https://www.mdpi.com/article/10.3390/nu17213334/s1. File S1: Preferred Reporting Items for Systematic reviews and Meta-Analyses extension for Scoping Reviews (PRISMA-ScR) Checklist; File S2: Search strategy.
