# Supplementary material for: Synergistic Effects of Exercise and Nano-Curcumin Supplementation in Women with Lifestyle-Related Diseases: A Scoping Review
Source: Nutrients. 2025 Oct 23;17(21):3334. doi: 10.3390/nu17213334 (PMC12609984; doi:10.3390/nu17213334)
Supplement: Supplementary file 1 [file nutrients-17-03334-s001.zip › nutrients-3920662-supplementary/Supplementary File S2 - Updated.pdf]

Synergistic Effects of Exercise and Nano-Curcumin Supplementation on Women with Lifestyle-Related Diseases: A Scoping Review

---

Search strategy and results

Web of Science = 22

(“nano-curcumin” OR “nano curcumin” OR “nanocurcumin”) (All Fields) and (“physical activit*” OR “exercis*” OR “exercise therap*” OR “training*” OR “physical fitness” OR “fitness” OR “physical exercis*” OR “strength training*” OR “endurance training*” OR “flexibility training*” OR “weight training*” OR “resistance training*” OR “circuit training” OR “meditation” OR “yoga”) (All Fields)

https://www.webofscience.com/wos/woscc/summary/b94a1f0c-5092-4c21-aa27-b972c7a35135-0168b2fb50/relevance/1

Scopus = 30

(TITLE-ABS-KEY ("nano-curcumin" OR "nano curcumin" OR "nanocurcumin")) AND (TITLE-ABS-KEY ("physical activit*" OR "exercis*" OR "exercise therap*" OR training* OR "physical fitness" OR "fitness" OR "physical exercis*" OR "strength training*" OR "endurance training*" OR "flexibility training*" OR "weight training*" OR "resistance training*" OR "circuit training" OR "meditation" OR "yoga"))

Pubmed = 18

("nano-curcumin"[All Fields] OR "nano-curcumin"[All Fields] OR "nanocurcumin"[All Fields]) AND ("physical activit*"[All Fields] OR "exercis*"[All Fields] OR "exercise therap*"[All Fields] OR "training*"[All Fields] OR "physical fitness"[All Fields] OR "fitness"[All Fields] OR "physical exercis*"[All Fields] OR "strength training*"[All Fields] OR "endurance training*"[All Fields] OR "flexibility training*"[All Fields] OR "weight training*"[All Fields] OR "resistance training*"[All Fields] OR "circuit training"[All Fields] OR "meditation"[All Fields] OR "yoga"[All Fields])

Other Sources = 71

Exercise, sports, training, physical activity, nano-curcumin, nano-curcumin, nanocurcumin

https://scholar.google.com/scholar?start=60&q=exercise+sports+training+physical+activity+nano-curcumin+nano-curcumin+nanocurcumin&hl=en&as_sdt=0,5
